# Prevalence of Infertility Problems among Iranian Infertile Patients Referred to Royan Institute

**DOI:** 10.22074/ijfs.2016.5043

**Published:** 2016-09-05

**Authors:** Mahdi Sepidarkish, Amir Almasi-Hashiani, Fatemeh Shokri, Samira Vesali, Elaheh Karimi, Reza Omani Samani

**Affiliations:** 1 Department of Epidemiology and Reproductive Health, Reproductive Epidemiology Research Center, Royan Institute for Reproductive Biomedicine, ACECR, Tehran, Iran; 2 Department of Epidemiology and Biostatistics, School of Public Health, Tehran University of Medical Sciences, Tehran, Iran

**Keywords:** Infertility, Assisted Reproductive Technology, Iran

## Abstract

**Background:**

Few studies have been conducted on the infertility problems in Iran. This study aimed to investigate the prevalence of infertility problems and related factors in Iranian infertile patients.

**Materials and Methods:**

In this cross sectional study, 405 infertile patients referred to Royan Institute, Tehran, Iran, between 2014 and 2015, were selected by simple random sampling. Participants completed the Fertility Problem Inventory (FPI) including 46 questions in five domains (social concern, sexual concern, relationship concern, rejection of parenthood, and need for parenthood). Mean difference between male and female was verified using independent-samples Student’s t test. A generalized linear model (GLM) was also used for testing the effect of variables on the fertility problems. Data was analyzed using Stata software version 13.

**Results:**

The mean age (SD) of participants was 31.28 (5.42). Our results showed that 160 infertile men (95.23%) were classified as very high prevalence of infertility problems. Among infertile women, 83 patients (35.02%) were as very high prevalence of infertility problems, and 154 patients (64.98%) were as high prevalence. Age (P<0.001), sex (P<0.001), a history of abortion (P=0.009), failure of previous treatment (P<0.001), and education (P=0.014) had a significant relationship with FPI scores.

**Conclusion:**

Bases on the results of current study, an younger male with lower education level, history of abortion and history of previous treatments failure experienced more infertility problems.

## Introduction

Infertility as a critical concept threatens individual stability and social relations (1). Although infertility is not considered as a medical issue, it critically affects the life of infertile couples in all aspects (2). Couples experiencing this crisis situation are more at risk for depression, anxiety, low self-esteem, and dissatisfaction (3). Infertile couples face both the physical and psychological problems during the diagnosis and treatment which can disrupt their lifestyle. Infertility can influence the relationship between the infertile patients with their spouse, friends, and colleagues, while it has a negative impact on their adherence performance (4, 5). Impulsive angry behaviors, feelings of helplessness and worthlessness, anxiety (particularly in the longterm treatments), concerns of sexual attraction, feeling of isolation, physical complaints, difficulty in sexual relationships, and sexual dissatisfaction are the problems reported by two recent studies (6, 7). 

The prevalence of problems associated with infertility has also been reported in other studies. However, according to studies by Ashkani et al. (8) and Fassino et al. (9), only 20 to 60 percent of infertile couples experienced these types of problems. There is a remarkable infertility rate in Iran (10). In Iranian culture, fertility is considered as an important concept. Unfortunately, few studies have been carried out on the different infertility problems, and most of them have focused on the psychological aspects of the subject (11, 12). Assessment of the problems related to infertility with a comprehensive tool is necessary (13). Therefore, this study aimed to investigate the prevalence of infertility problems and related factors in Iranian infertile patients.

## Materials and Methods

This cross-sectional study was performed on 405 infertile patients referred to Royan Institute, Tehran, Iran, between 2014 and 2015. Simple random sampling was applied using a random numbers table based on the medical records of patients in this center. Inclusion criteria were as follows: definitive confirmation of infertility (primary or secondary) by a reproductive specialist, male factor infertility/ female factor infertility (or both), unexplained in- fertility, and willingness to participate in the study. The diagnosis of infertility, cause of infertility, and infertility were defined based on the recommendations of World Health Organization (WHO) as follows: clinical infertility is defined as the lack of clinical pregnancy after 12 months of regular intercourse to have children. No history of previous pregnancy as primary infertility and secondary infertility was defined as a couple who experienced successful fertility in the past, and face infertility after previous pregnancies. A phone call was made to all infertile patients using simple random sampling, as mentioned. In a meeting with the patients, purpose of the study and the confidentiality of the data were clearly explained. Eligible individuals were also assured that acceptance or refusal to participate in the research had no influence on their treatment procedures. Filling the questionnaire was considered as signing a written informed consent. The study was approved by the Ethics Committee of Royan Institute. Participants completed two questionnaires. Firstly, a questionnaire about the socio-demographic and clinical characteristics was filled out that includes age (years), sex (male or females), educational levels (primary, secondary, and university), duration of infertility (years), type of infertility (primary, secondary), duration of marriage (years), cause of infertility (male factor, female factor, both, or unexplained infertility), the number of previous abortion (gestational age <20 weeks), failure of previous treatment (0,1, 2, 3, ≥4), and a history of abortion (yes, no). Secondly, the Fertility Problem Inventory (FPI) was completed. This questionnaire, which was made by Newton in 1999 to detect problems associated with infertility, includes 46 questions and five domains (social concern, sexual concern, relationship concern, rejection of parenthood, and need for parenthood). It is a 6-point Likert scale, from strongly disagree=1 to strongly agree=6. In this tool, 19 questions are scored inversely. The scores are between 46 and 276, indicating that the highest numerical values represent very high prevalence of problems related to infertility. In men, the scores range from 0 to 87 as less prevalence, 88 to 113 as moderate prevalence, 114 to 146 as high prevalence, and more than 147 as very high prevalence. In women, scores 0 to 97 are classified as low prevalence, 98 to 132 as moderate prevalence, 133 to 167 as high prevalence, and over 168 as very high prevalence (13). In a study by Ramezanzadeh et al. (6), the validity and reliability of the Iranian version of FPI were evaluated in Iranian infertile patients and Cronbach’s alpha coefficient was greater than 70% for all domains.

### Statistical analysis

Categorical data were presented as numbers (percent) and continuous data as mean (SD). Mean difference between male and female was verified by independent-samples Student’s t test. A generalized linear model (GLM, family: Gaussian, link: identity) was used to test the effect of age, sex, educational level, duration of infertility (years), cause of infertility, type of infertility, failure of previous treatment, and history of abortion on the fertility problem score. Results were presented as standardized coefficients with 95 percent confidence intervals (CIs). Values of P less than 0.05 were considered statistically significant. Data was analyzed using Stata software (StataCorp, USA).

## Results

A group of 410 participants completed both questionnaires. Five questionnaires (1.21%) were excluded due to the completion rate of less than 70%. The mean (SD) age was 31.28 (5.42). Sociodemographic and clinical characteristics of the participants are shown in the Table 1. 

**Table 1:** Socio-demographic and clinical characteristics of the participants


Age (Y)	31.28 ± 5.42
Duration of infertility (Y)	4.93 ± 4.01
Duration of marriage (Y)	7.07 ± 4.23
Number of previous abortion	1.04 ± 0.87
Sex	
Male	168 (41.5)
Female	237 (58.5)
Cause of infertility	
Male factor	146 (36)
Female factor	88 (21.7)
Both	71 (17.5)
Unexplained	100 (24.8)
Type of infertility	
Primary	287 (70.9)
Secondary	118 (29.1)
Educational level	
Primary	90 (22.2)
Secondary	150 (37)
University	165 (40.8)
Failure of previous treatment	
0	208 (51.35)
1	81 (20)
2	61 (15.06)
3	36 (8.88)
≥4	19 (4.69)
History of abortion	
No	316 (78)
Yes	89 (22)


Values are given as mean ± SD or number (percentage) unless otherwise indicated.

Table 2 shows the mean scores of FPI domains compared between males and females. Our findings showed that a very high prevalence of fertility problems in infertile patients, meaning among infertile men, 160 individuals (95.23%) were classified as very high prevalence and the remaining 8 individuals (4.77%) as high prevalence. None of them were as less or moderate prevalence. Among infertile women, 83 patients (35.02%) were as very high prevalence and 154 (64.98%) as high prevalence. Like infertile men, no women were as less or moderate prevalence.

The GLM results showed that five out of eight predictors were statistically significant. The model was statistically significant [F (8, 397) =11.13, P<0.001] and accounted for approximately 16% of the variance of scores (R^2^=0.185, Adjusted R^2^=0.168). Basic descriptive statistics, crude and standardized regression coefficients are shown in Table 3. The strongest effect belongs to age followed by sex, a history of abortion, failure of previous treatment, and education. The results suggested that patients who had a history of abortion showed very high prevalence of fertility problem as compared to patients without a history of abortion (P<0.001). Similarly, patients with a history of failure in previous treatments had very high prevalence of fertility problems (P=0.001). As seen in Table 3, age was a significant predictor of prevalence of fertility problems. When the other predictors were ignored, age was negatively correlated with prevalence of fertility problem, meaning that an increase of one SD of age resulted in a decrease of 0.231 in mean scores of FPI. Like crude analysis, female had lower mean scores of FPI as compared to the related value of male. Also, mean scores of FPI decreased with an increase in the education level. 

**Table 2:** Comparison of the mean scores of fertility problem inventory domains between males and females


Scale	Total (n=405)	Male (n=168)	Female (n=237)	P value

Social concern	34.36 ± 5.46	34.05 ± 5.64	34.58 ± 5.32	0.345
Sexual concern	29.82 ± 4.35	31.11 ± 4.02	28.9 ± 4.35	<0.001
Relationship concern	36.9 ± 3.58	36.81 ± 3.54	36.96 ± 3.61	0.678
Rejection of parenthood	25.66 ± 5.3	27.46 ± 5.56	24.38 ± 4.71	<0.001
Need for parenthood	37.37 ± 5.09	36.37 ± 6	38.08 ± 4.2	0.002
All scales	164.12 ± 10.42	165.82 ± 11.27	162.91 ± 9.61	0.007


Values are given as mean ± SD.

**Table 3:** GLM results for FPI scores


Variables	Standardized coefficient β	Unstandardized coefficient β	95% CIs for β		P value
Lower bound	Upper bound

Age (Y)	-0.231	-0.426	-0.631	-0.222	<0.001
Sex	-0.225	-4.72	-6.82	-2.63	<0.001
Education	-0.12	-0.951	-1.71	-0.191	0.014
Failure of previous treatment	0.155	3.27	1.31	5.23	0.001
History of abortion	0.181	4.6	1.13	8.06	0.009


All P values are reported as two-tailed. Significance is defined as P<0.05. CIs; Confidence intervals, GLM; Generalized linear model, and FPI; Fertility problem inventory.

## Discussion

Our findings revealed that the prevalence of infertility problems is very high in infertile patients. In our study, 95.23% of male patients and 35.02% of female patients showed very high prevalence of infertility problems, so none of them was classified as less or moderate prevalence. After adjusting confounder variables (including age, sex, educational level, failure of previous treatment, and a history of abortion), a significant increase in prevalence of infertility problems was seen in younger male patients with lower education level, a history of previous abortion, and failure of previous treatment. 

Considering the cause of infertility, our findings showed that the male factor (36%) was more prevalent than the female factor (21.7%). In a study by Kamali et al. (14), they have found that 50.5% of participants suffered from the male factor, while 28.6% had the female factor. In another study by Noorbala et al. (15) they have reported prevalence values of 44 and 28% for psychiatric disorders in infertile and fertile women, respectively. Furthermore, the most important stress factors in infertile women were feedback from others (81.3%), loneliness (74%), treatment of infertility (60.7%), incomplete families (53%) and the identity disorder (50.7%).

In a study by Hussain et al. (16) on psychological disorders among women with polycystic ovary syndrome (PCOS), as one of the major causes of infertility, they have reported a prevalence of 23% for major depression in women with PCOS. They have also reported 15.4% suffering from panic disorder and 15.4% diagnosed with generalized anxiety disorder. The prevalence of suicide is also more among women with PCOS than others (16, 17). In a study by Ramazanzadeh et al. (6) on 370 infertile women, they have found that the prevalence values of depression and anxiety in infertile women were 40.8 and 86.8%, respectively. These findings were consistent with our results. Furthermore, the prevalence of psychiatric disorders in Iranian infertile women was higher as compared with the Western countries (15, 18). Odden et al. (19) have reported that the prevalence of 35.2% for psychiatric disorders in infertile women.

In our relationship between the age and total scores of FPI, suggesting, as age increased, the total scores decreased. It means that younger infertile patients experienced very high prevalence of problems related to infertility as competed to older infertile patients. Ramazanzadeh et al. (6) have also found that the prevalence of anxiety and depression is more common in 6-4 years after infertility. Another study has indicated that anxiety and depression improve with increasing age.

Various researches have showed different findings about the relationship between prevalence of infertility problems with age and education level. In a study by Beutel et al. (20), there was a significant correlation between prevalence of infertility problems with age and education level, while such a relationship was reported by two other studies (19, 21). In our study, the prevalence of infertility problems was significantly higher in the participants with a history of previous treatment failure. This may be due to the failure of fertility treatment, medical expenses, and the reaction of others. A history of abortion was known as one of the factors in prevalence of infertility problems. One of possible limitations of this study was the patients with more complex infertility problems who are mostly referred to Royan Institute from other centers. These patients are more likely to experience longer exposure to the problems related to infertility. Therefore, our findings can only be generalized to patients with more complex infertility problems referred to a fertility center. Finally, in all cross-sectional study, such as our study, there is no temporality. Therefore, determination of the exposure and the outcome is not easily performed and causality will be not detected. 

## Conclusion

The results showed that a very high prevalence of fertility problems in infertile couples. Bases on the results of current study, a younger male with lower education level, history of abortion and history of previous treatments failure experienced high prevalence of infertility problems.
